# Supplementary material for: Dynamic SARS-CoV-2 surveillance model combining seroprevalence and wastewater concentrations for post-vaccine disease burden estimates
Source: Commun Med (Lond). 2024 Apr 9;4:70. doi: 10.1038/s43856-024-00494-y (PMC11004132; doi:10.1038/s43856-024-00494-y)
Supplement: Supplementary file 2 — Reporting Summary [file 43856_2024_494_MOESM2_ESM.pdf]

nature portfolio

Corresponding author(s): Aruni Bhatnagar

Last updated by author(s): Jan 22, 2024

Reporting Summary

Nature Portfolio wishes to improve the reproducibility of the work that we publish. This form provides structure for consistency and transparency in reporting. For further information on Nature Portfolio policies, see our Editorial Policies and the Editorial Policy Checklist.

Statistics

For all statistical analyses, confirm that the following items are present in the figure legend, table legend, main text, or Methods section.

n/a	Confirmed	
☐	☒	The exact sample size (*n*) for each experimental group/condition, given as a discrete number and unit of measurement
☐	☒	A statement on whether measurements were taken from distinct samples or whether the same sample was measured repeatedly
☐	☒	The statistical test(s) used AND whether they are one- or two-sided<br>*Only common tests should be described solely by name; describe more complex techniques in the Methods section.*
☐	☒	A description of all covariates tested
☐	☒	A description of any assumptions or corrections, such as tests of normality and adjustment for multiple comparisons
☐	☒	A full description of the statistical parameters including central tendency (e.g. means) or other basic estimates (e.g. regression coefficient) AND variation (e.g. standard deviation) or associated estimates of uncertainty (e.g. confidence intervals)
☒	☐	For null hypothesis testing, the test statistic (e.g. *F*, *t*, *r*) with confidence intervals, effect sizes, degrees of freedom and *P* value noted<br>*Give P values as exact values whenever suitable.*
☐	☒	For Bayesian analysis, information on the choice of priors and Markov chain Monte Carlo settings
☐	☒	For hierarchical and complex designs, identification of the appropriate level for tests and full reporting of outcomes
☐	☒	Estimates of effect sizes (e.g. Cohen's *d*, Pearson's *r*), indicating how they were calculated

Our web collection on statistics for biologists contains articles on many of the points above.

Software and code

Policy information about availability of computer code

Data collection

Seroprevalence
Community-wide stratified randomized seroprevalence sampling (Supplementary Table 1) was conducted in four waves from April to August 2021 in Jefferson County, Kentucky (USA) which is also the consolidated government for the city of Louisville.14 Seroprevalence sampling was conducted both before and during vaccination, but this analysis only considers the period after COVID-19 vaccines were made widely available to the public (N = 3,303). In some cases, due to the timing of sampling waves, respondents may have had only the first of a two-dose vaccine series. Serological positivity for nucleocapsid immunoglobulin G was used to identify participants with previous SARS-CoV-2 natural infection; vaccines used in the studied areas rely on SARS-CoV-2 viral spike protein and thus spike protein presence could be attributable to either natural infection or vaccination. Owing to elevated levels of vaccinated respondents in our study (~90%), we only included seroprevalence measured by response to IgG N1 antibodies.14,15 The nucleocapsid (N1) IgG test sensitivity was 65% and the specificity was 85%.14 It was assumed over the study period vaccination induced antibodies did not decay below detection.

Concentration of SARS-CoV-2 and PMMoV in the wastewater
Wastewater samples were collected twice per week from five wastewater treatment plants (N = 168; Supplementary Figure 1 and Supplementary Table 2) from April to August 2021. From an influent 24-hour composite sampler, 125 ml of subsample was collected and analyzed for SARS-CoV-2 (N1) and PMMoV. In a few cases due to an equipment malfunction, a grab sample was collected. The geographic area and population serviced by a wastewater treatment plant comprises a sewershed, the zone for which we consider in our model analysis across a range of population sizes, income levels and racial and ethnic diversity. Analysis used polyethylene glycol (PEG) precipitation with quantification in triplicate by reverse transcription polymerase chain reaction (RT-qPCR).2 Data for SARS-CoV-2 (N1) and PMMoV are reported as weekly average copies/ml of wastewater with a threshold value for SARS-CoV-2 (N1) assays of 7.5 copies/ml and for PMMoV 143 copies/ml.

Administrative COVID-19 data

Administrative data on COVID-19 vaccination and infected individuals' hospitalization was provided by the Jefferson County health authority, Louisville Metro Department of Public Health and Wellness (LMPHW), under a Data Transfer Agreement. Vaccination data were geocoded to the urban sewersheds using ArcGIS Pro version 2.8.0 (Redlands, CA). Daily hospitalization data was only available aggregated at a county level.

Data analysis

Analytical model
The hybrid model for estimating the effect of vaccination and variants on longitudinal wastewater concentration was developed by combining a compartmental ecological model with a statistical linear model (Supplemental Information). The former was used to longitudinally estimate population prevalence from the observed cross-sectional rates of seropositivity. We assumed the overall vaccination pattern as reported by the county, with the overall adult vaccination rate reaching 64%16 by the end of the study period. The hybrid model was used to relate the ecological model prevalence to the wastewater concentration. The ecological model, SVI_2 RT, tracked longitudinally the proportions of individuals who were susceptible (S), vaccinated (V), infected with non-Delta variant (I_1), infected with Delta variant (I_2), recovered (R), or seropositive (T). We note that a version of this model that did not account for vaccination or variant was considered in our earlier work.2

Upon estimating the parameters in the SVI_2 RT model, we compared the model-calculated prevalence estimates for SARS-CoV-2 infections and vaccination levels with the wastewater concentration levels of SARS-CoV-2 (N1) and for that normalized by PMMoV.17 We also separately calculated two prevalence estimates according to the Alpha and Delta variants. Bayesian linear regression was performed both on the county aggregated data and stratified by sub-county wastewater treatment plant zones (sewersheds). We used the broken stick regression model to separately compare the Alpha and Delta variation effects on the wastewater concentration with regression coefficients directly. To improve the regression model stability, we used weekly average prevalence rates from the SVI_2 RT model as the explanatory variable, and weekly aggregated average wastewater concentrations as the single outcome variable. This temporal aggregation also allowed us to use a simple posterior-profile likelihood to estimate the average change point in the broken stick regression model (see, e.g., Schwartz et al.18 for a similar approach for initial conditions imputation). We assigned non-informative priors to all regression parameters. Specifically, the non-informative Cauchy distribution was assigned to regression coefficients, and the non-informative gamma prior was assigned to the dispersion parameter in error term. The regression model with intercept is used where the intercept may be interpreted as background and calibration noise related to wastewater sampling. We could see temporal differences between the Alpha and Delta variant dominant dates (Supplementary Table 3), but this variability in time also considers that samples are weekly aggregated average wastewater concentrations. We did not include these variabilities of intervals in the model as the magnitudes of the observed wastewater concentration and estimated prevalence in this interval are relatively small, and model changes do not alter the overall model fit.

The strong statistical significance of the regression model relating prevalence and wastewater concentration allowed for indirect estimation of the effect of population vaccination and variants. Under the assumption the relationship between the wastewater concentration and the prevalence is not confounded by the vaccination and variants, we used the original regression equation derived from the collected wastewater and seroprevalence data to estimate the wastewater concentration over time. To estimate the vaccination effect, we compared these concentrations with hypothetical ones obtained when the vaccination term was zeroed out in the SVI_2 RT model. In a comparable manner, we estimated the effect of the introduction of the Delta variant. Finally, we performed the longitudinal, regression-based analysis relating the community hospitalization to observed wastewater concentrations. In the three analyses we quantified the effects by calculating the size of the effects relative to the factual (observed) states.

Wastewater samples were prepared for whole genome sequencing,19,20 and the proportion of observed SARS-CoV-2 variants was estimated for each sewershed based on variant dominance (Supplementary Figure 2 and Supplementary Table 3). Two variants were present in the study area during the study period: Alpha was dominant from April until July, while Delta was dominant from July until August.19,20 To reflect the infections before and after the emergence of the Delta variant, we incorporated into our SVI2RT model the two different infection compartments (I_1 and I_2) reflecting both the infection competition and temporal heterogeneity caused by two different variants of the virus.

For manuscripts utilizing custom algorithms or software that are central to the research but not yet described in published literature, software must be made available to editors and reviewers. We strongly encourage code deposition in a community repository (e.g. GitHub). See the Nature Portfolio guidelines for submitting code & software for further information.

Data

Policy information about availability of data

All manuscripts must include a data availability statement. This statement should provide the following information, where applicable:

- Accession codes, unique identifiers, or web links for publicly available datasets
- A description of any restrictions on data availability
- For clinical datasets or third party data, please ensure that the statement adheres to our policy

The seroprevalence data, wastewater levels, and hospitalization information utilized in this study, along with the computer code employed for the analysis, are accessible on GitHub. You can find the complete set of data and code at https://github.com/cbskust/DSA_Seroprevalence.

Human research participants

Policy information about studies involving human research participants and Sex and Gender in Research.

Reporting on sex and gender	Information not reported.
Population characteristics	See above.
Recruitment	See above.

Ethics oversight	For the seroprevalence and data provided by the LMPHW under a Data Transfer Agreement, the University of Louisville Institutional Review Board approved this as Human Subjects Research (IRB number: 20.0393). For the wastewater data, the University of Louisville Institutional Review Board classified this as non-human subjects research (reference #: 717950).

Note that full information on the approval of the study protocol must also be provided in the manuscript.

Field-specific reporting

Please select the one below that is the best fit for your research. If you are not sure, read the appropriate sections before making your selection.

☒ Life sciences ☐ Behavioural & social sciences ☐ Ecological, evolutionary & environmental sciences

For a reference copy of the document with all sections, see nature.com/documents/nr-reporting-summary-flat.pdf

Life sciences study design

All studies must disclose on these points even when the disclosure is negative.

Sample size	Previously published as: Keith RJ, Holm RH, Amraotkar AR, Bezold MM, Brick JM, Bushau-Sprinkle AM, Hamorsky KT, Kitterman KT, Palmer KE, Smith T, Yeager R, Bhatnagar A. Stratified Simple random sampling versus volunteer community-wide sampling for estimates of COVID-19 prevalence. American Journal of Public Health 2023;113(7):768-777.
Data exclusions	Provided in<br>Appendix C. Population vaccination model (SVI2RT)
Replication	Provided in<br>Appendix C. Population vaccination model (SVI2RT)
Randomization	Randomization was not relevant.
Blinding	Blinding was not relevant.

Reporting for specific materials, systems and methods

We require information from authors about some types of materials, experimental systems and methods used in many studies. Here, indicate whether each material, system or method listed is relevant to your study. If you are not sure if a list item applies to your research, read the appropriate section before selecting a response.

Materials & experimental systems

n/a	Involved in the study	
☐	☒	Antibodies
☒	☐	Eukaryotic cell lines
☒	☐	Palaeontology and archaeology
☒	☐	Animals and other organisms
☐	☒	Clinical data
☒	☐	Dual use research of concern

Methods

n/a	Involved in the study	
☒	☐	ChIP-seq
☒	☐	Flow cytometry
☒	☐	MRI-based neuroimaging

Antibodies

Antibodies used	Community-wide stratified randomized seroprevalence sampling (Supplementary Table 1) was conducted in four waves from April to August 2021 in Jefferson County, Kentucky (USA) which is also the consolidated government for the city of Louisville.14 Seroprevalence sampling was conducted both before and during vaccination, but this analysis only considers the period after COVID-19 vaccines were made widely available to the public (N = 3,303). In some cases, due to the timing of sampling waves, respondents may have had only the first of a two-dose vaccine series. Serological positivity for nucleocapsid immunoglobulin G was used to identify participants with previous SARS-CoV-2 natural infection; vaccines used in the studied areas rely on SARS-CoV-2 viral spike protein and thus spike protein presence could be attributable to either natural infection or vaccination. Owing to elevated levels of vaccinated respondents in our study (~90%), we only included seroprevalence measured by response to IgG N1 antibodies.14,15 The nucleocapsid (N1) IgG test sensitivity was 65% and the specificity was 85%.14 It was assumed over the study period vaccination induced antibodies did not decay below detection.
Validation	Previously published as: Keith RJ, Holm RH, Amraotkar AR, Bezold MM, Brick JM, Bushau-Sprinkle AM, Hamorsky KT, Kitterman KT,

Validation	Palmer KE, Smith T, Yeager R, Bhatnagar A. Stratified Simple random sampling versus volunteer community-wide sampling for estimates of COVID-19 prevalence. American Journal of Public Health 2023;113(7):768-777.

Clinical data

Policy information about clinical studies

All manuscripts should comply with the ICMJE guidelines for publication of clinical research and a completed CONSORT checklist must be included with all submissions.

Clinical trial registration	Not a clinical trial
Study protocol	Previously published as: Keith RJ, Holm RH, Amraotkar AR, Bezold MM, Brick JM, Bushau-Sprinkle AM, Hamorsky KT, Kitterman KT, Palmer KE, Smith T, Yeager R, Bhatnagar A. Stratified Simple random sampling versus volunteer community-wide sampling for estimates of COVID-19 prevalence. American Journal of Public Health 2023;113(7):768-777.
Data collection	April 2021-August 2021
Outcomes	We used weekly SARS-CoV-2 wastewater concentration with a stratified random sampling of seroprevalence, and spatially linked vaccination and hospitalization data, from April 2021-August 2021.
